# Resistance to thiophanate‐methyl and azoxystrobin in *Colletotrichum* species from Brazilian grapes

**DOI:** 10.1002/ps.71203

**Published:** 2026-08-17

**Authors:** Daniel Ricardo Maass Steiner, Camila Iavorski Zela, Debora Franco de Oliveira, Jhulia Gelain, Guido Schnabel, Louise Larissa May De Mio

**Affiliations:** ^1^ Plant Science and Plant Health Department Federal University of Paraná Curitiba Brazil; ^2^ Department of Plant and Environmental Sciences Clemson University Clemson SC USA

**Keywords:** *β‐tubulin* gene, *cytb* gene, grape ripe rot, MBC, QoI, resistance

## Abstract

**BACKGROUND:**

Grape ripe rot (GRR) caused by *Colletotrichum* species can cause yield loss in vineyards in Southern and Northeast Brazil. Thiophanate‐methyl (FRAC 1) and azoxystrobin (FRAC 11) fungicides are frequently used to control GRR, but resistance to these fungicides in *Colletotrichum* species may impact their efficacy.

**RESULTS:**

Sensitivity to thiophanate‐methyl was assessed for 89 isolates of the *C*. *gloeosporioides* species complex using mycelial growth inhibition. Isolates were classified as sensitive (*n* = 2), low‐resistant (*n* = 70) or highly resistant (*n* = 17). Highly resistant isolates had nucleotide mutations at amino acid codons 198 and 200 in the *β*‐*tubulin* gene. Sensitivity to azoxystrobin was assessed for 176 isolates belonging to the *C*. *gloeosporioides* and *C*. *acutatum* species complexes using conidial germination inhibition. Of these, 33 isolates were resistant, and salicylhydroxamic acid (SHAM) was required to detect those carrying the G143A amino acid substitution in the cytochrome *b* (*cytb*) gene. Detached fruit assays revealed that thiophanate‐methyl and azoxystrobin applied at label rates were not effective against highly resistant and resistant isolates, respectively. Seven *Colletotrichum* spp. isolates were resistant to both fungicides.

**CONCLUSION:**

This study is the first to report *Colletotrichum* spp. isolates from Brazilian vineyards resistant to azoxystrobin, thiophanate‐methyl, or both. Resistance was associated with *β‐tubulin* (E198A, F200Y) and *cytb* (G143A) mutations. These results demonstrate the risk of control failure with FRAC 1 and 11 fungicides and highlight the need for resistance management strategies in vineyards. © 2026 The Author(s). *Pest Management Science* published by John Wiley & Sons Ltd on behalf of Society of Chemical Industry.

## Graphical abstract

First report of resistance to both quinone outside inhibitor (QoI; azoxystrobin) and methyl benzimidazole carbamate (MBC; thiophanate‐methyl) fungicides in *Colletotrichum* isolates associated with grapevine in Brazil.

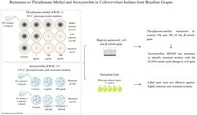

## 1 INTRODUCTION

Grape ripe rot (GRR), caused by *Colletotrichum gloeosporioides* (*C*.*g*. complex), *C*. *acutatum* (*C*.*a*. complex) and *C*. *boninense* species complexes, ranks among the three most destructive grapevine diseases in Brazil.^1^, ^2^, ^3^ The pathogen can infect mature berries during frequent rains, high humidity and warm temperatures, leading to significant losses.^4^ The disease was first documented in São Paulo in the 1800s, later in Southern Brazil in the 1960s, and more recently (2021) in Northeast Brazil.^4^ This steady spread has raised concerns among Brazilian growers since the early 2000s.^5^ One technical report highlights GRR outbreaks in the south and northeast of Brazil, the two main grape‐producing regions in the country.^4^

Grape farmers commonly apply multisite fungicides such as mancozeb, alongside site‐specific fungicides like quinone outside inhibitors (QoIs; FRAC 11) and methyl benzimidazole carbamates (MBCs; FRAC 1) for GRR management.^6^ Frequent use of fungicides with site‐specific modes of action can favor the selection of resistant phenotypes.^7^

Azoxystrobin has been available for more than 20 years and has been widely used by Brazilian growers for the control of various plant diseases.^8^ It belongs to the QoI fungicide group and acts by interfering with mitochondrial respiration and energy production processes.^9^ The resistance is qualitative, with the predominant mutation being G143A. Other point mutations, including F129L and G137R, usually confer moderate resistance.^10^, ^11^, ^12^ QoIs block ATP production by binding to the Qo site of the cytochrome *b* (cyt*b*) protein in complex III of the mitochondrial respiratory chain.^13^ In *in vitro* assays assessing sensitivity to QoI fungicides, salicylhydroxamic acid (SHAM) is often added to inhibit alternative respiration.^14^, ^15^ The latter is activated by an alternative oxidase (AOX)‐mediated pathway, allowing continued energy production and resulting in conidial germination or mycelial growth on QoI‐amended media, which may lead to false‐positive resistance.^14^, ^15^, ^16^ By inhibiting AOX, SHAM helps ensure a more accurate evaluation of QoI sensitivity; although, for grapevine‐associated *Colletotrichum* spp., the necessity of SHAM inclusion in *in vitro* assays has not yet been studied.

Thiophanate‐methyl is an MBC fungicide that acts upon the β‐tubulin subunits, hindering the formation of microtubules.^17^ Point mutations in the target gene *β‐tubulin* (F167Y, E198A, E198K, E198G and F200Y) constitute the main mechanism of resistance.^18^ Thiophanate‐methyl is effective against isolates belonging to the *C*.*g*. complex, whereas isolates from the *C*.*a*. complex are often reported to be inherently insensitive to MBC fungicides.^19^

In Brazil, cases of resistance to MBC and QoI fungicides exist in *Colletotrichum* species affecting strawberries, and soybean, among others.^20^, ^21^ However, *Colletotrichum* spp. of grapevine in Brazil have not been investigated. The objectives of this study were to: (i) determine the sensitivity of *Colletotrichum* spp. isolates to thiophanate‐methyl and azoxystrobin, (ii) clarify the need for SHAM in *in vitro* assays to determine azoxystrobin sensitivity, (iii) investigate *β*‐*tubulin* and *cytb* gene sequences in resistant isolates, and (iv) assess efficacy of label rates of formulated products against the resistance phenotypes identified in this study.

## 2 MATERIALS AND METHODS

### 2.1 Fungal isolates and culture conditions

The isolates were obtained from grapevine asymptomatic tissues (leaves, flowers and fruits) and from ripe rot lesions previously identified to the species level using phylogenetic analyses.^1^ Isolates from the *C*.*a*. complex were 74 *C*. *nymphaeae*, 8 *C*. *melonis* and 5 *C*. *cf*. *melonis*; and from the *C*.*g*. complex were 30 *C*. *siamense*, 29 *C*. *viniferum*, 18 *C*. *chrysophilum*, 3 *C*. *fructicola*, 3 *C*. *jiangxiense*, 3 *C*. *hebeiense*, 2 *C*. *theobromicola* and 1 *C*. *gloeosporioides*. Isolates were collected from Rio Grande do Sul (6 isolates), Paraná (147 isolates), São Paulo (1 isolate), Distrito Federal (8 isolates), Pernambuco (12 isolates) and Bahia (2 isolates) states between 2022 and 2023. Isolates were single‐spored and stored on filter paper at –20 °C in the culture collection of the Epidemiology Laboratory for Integrated Disease Management (LEMID) at the Federal University of Paraná. For the *in vitro* and *in vivo* assays, isolates were grown on Potato Dextrose Agar medium (PDA) at 25 °C under constant light for 5–7 days.

### 2.2 Fungicide sensitivity assays

Conidial germination was assessed to determine QoI sensitivity in isolates from the *C*.*a*. complex (87 isolates) and *C*.*g*. complex (89 isolates) using azoxystrobin (Amistar WG®, 50% a.i.; Syngenta Crop Protection, São Paulo/SP, Brazil). A 5‐mL volume of autoclaved water was transferred to each isolate colony plate, and using a Drigalski loop, fungal colonies were scraped to obtain the spore suspension. The suspension was then filtered through sterile gauze, and the spore suspension was adjusted to 2 × 10^4^ conidia/mL. A total of 100 μL of the suspension was added to PDA containing fungicide concentrations of 3 and 100 μg mL^−1^. Isolates whose conidial germination was inhibited at 3 μg mL^−1^ were classified as sensitive. Isolates not inhibited at 3 μg mL^−1^ but inhibited at 100 μg mL^−1^ were classified as moderately resistant, whereas those not inhibited at 100 μg mL^−1^ were considered resistant.^22^ All isolates were tested with and without SHAM at 100 μg mL^−1^ and three replicates were used for each treatment.^23^ For each replicate, the germination of 100 conidia was evaluated. The experiment was conducted twice. The sensitivity of 89 *C*.*g*. complex isolates to thiophanate‐methyl (Cercobin 700 WG, 70% a.i. Ihara, Sorocaba/SP, Brazil) was determined using discriminatory doses of 0, 1, 100 and 500 μg mL^−1^ (adapted from refs^24^, ^25^) and the mycelium diameter was measured after 3 days. The percentage of mycelial growth inhibition (GI) was calculated using the formula: ((Control − Treatment)/Control) × 100). Isolates were classified as sensitive (GI ≥ 90% at 1 μg mL^−1^), low‐resistant (GI < 90% at 1 μg mL^−1^ and GI ≥ 90% at 100 μg mL^−1^) and highly resistant (GI < 90% at 100 μg mL^−1^ and 500 μg mL^−1^).^24^

### 2.3 Sequencing of the partial *β‐tubulin* and *cytb* genes

Four MBC‐highly resistant, two sensitive and two low‐resistant *C*.*g*. complex isolates were chosen for *β‐tubulin* sequence analysis and 10 QoI‐resistant and 16 sensitive isolates were chosen for *cytb* gene analysis. Genomic DNA was extracted from 7‐day‐old colonies cultured on PDA.^26^ Polymerase chain reactions (PCR) were performed using the GoTaq® Green Master Mix with a final volume of 25 μL, containing 1.0 μL of each primer at 10 nm, 1 μL DNA template at between 50 and 100 ng μL^−1^, 12.5 μL GoTaq® Green Master Mix and 9.5 μL nuclease‐free water. The *β*‐*tubulin* gene fragment was amplified using primers TB2L (5′‐GYTTCCAGATYACCCACTCC‐3′) and TB2R (5′‐TGAGCTCAGGAACRCTGACG‐3′).^27^ For *C*.*a*. complex isolates, the *cytb* gene fragment was amplified using the primers C. gramcytb‐bf1 (5′‐GAAGAGGTATGTACTACGGTTCATATAG‐3′) and C. gramcytb‐br1 (5′‐TAGCAGCTGGAGTTTGCATAG‐3′).^22^ For *C*.*g*. complex isolates, the *cytb* gene was amplified using the primers Cocytb‐F2 (TTACTTGCAGTTTGTTTAGG) and Cocytb‐R3 (ACCAGTGACCCAGTCTGTA) for the F129L mutation, and Cocytb‐F3 (TCTATTACAGAAGCAAGTACAG) and Cocytb‐R4 (ACTCAACGATATCTTGTCCA) for the G143A mutation.^28^ The PCR products were purified using DNA Clean & Concentrator (Zymo Research, CA, USA) and submitted for Sanger sequencing (Eurofins, Louisville, KY, USA). All sequences obtained were deposited in the GenBank database.

The following sequences from GenBank were included as references: *C*. *siamense* highly resistant to MBC fungicides (KM885297),^23^ *C*. *cereale* resistant to MBC fungicides (FJ476051),^29^ *C*. *siamense* sensitive to MBC fungicides (KM885294),^23^ *C*. *siamense* resistant to QoI fungicides (KX885103),^30^ *C*. *acutatum* sensitive to QoI fungicides (MF784586.1),^22^ and QoI‐resistant *C*. *siamense* (KM885303)^23^ and *C*. *acutatum* (MF784587.1).^22^

### 2.4 Detached fruit study

The efficacy of the label rates of formulated azoxystrobin (Amistar WG®, 50% a.i.; Syngenta Crop Protection) and thiophanate‐methyl (Cercobin 700 WG, 70% a.i.; Ihara) was evaluated on grape berries using QoI‐sensitive and QoI‐resistant isolates, as well as MBC‐sensitive, low‐resistant and highly resistant isolates. For thiophanate‐methyl, isolates of *C*. *theobromicola*, *C*. *fructicola*, *C*. *chrysophilum*, *C*. *hebeiense*, *C*. *viniferum* and *C*. *siamense* were included, comprising 2 sensitive, 6 low‐resistant and 11 highly resistant isolates. For azoxystrobin, isolates of *C*. *nymphaeae*, *C*. *gloeosporioides*, *C*. *fructicola*, *C*. *chrysophilum*, *C*. *hebeiense*, *C*. *viniferum* and *C*. *siamense* were evaluated, including 10 sensitive and 12 resistant isolates. *Vitis vinifera* (cv: Thompson) were disinfected in 0.7% sodium hypochlorite for 6 min, then rinsed twice with sterile deionized water. The experiment followed an isolate × wounding treatment design (wounded and non‐wounded berries). The experiment followed a 2 × 2 factorial design consisting of wounding treatment (wounded and non‐wounded berries) and fungicide treatment (with and without fungicide). For each isolate, three wounded and three non‐wounded berries were placed in each box. Each treatment combination consisted of three replicates with three berries each, totaling nine berries per treatment: wounded + fungicide, wounded without fungicide, non‐wounded + fungicide, and non‐wounded without fungicide (negative control). Thus, a total of 36 berries were used per isolate. The berries were then placed in plastic boxes containing two autoclaved filter paper sheets and surface‐dried inside a laminar flow hood for 20 min. The filter papers were then moistened with 50 mL of deionized water. The two filter paper sheets placed at the bottom of the boxes absorbed the water that had been added, remaining only moist. No free water was in contact with the grape berries. The experiment was conducted twice for all isolates.

Fruit were sprayed to run‐off with label rates of fungicide using a hand mister and incubated in lidded boxes at room temperature for 24 h before inoculation. Control berries were sprayed with autoclaved deionized water. A 5‐μL droplet of a 10^6^ conidia/mL suspension of each *Colletotrichum* isolate was placed on each berry and berries were incubated in closed boxes at 22–26 °C for a further 7 days and then assessed for GRR incidence.

Disease incidence was calculated as the proportion of infected fruit per experimental unit and expressed as a percentage. Analyses were performed in R (R Core Team).^31^

## 3 RESULTS

### 3.1 Sensitivity of *Colletotrichum* spp. isolates from grapevine to the fungicide thiophanate‐methyl and azoxystrobin

Based on the *in vitro* assays, most isolates (70) exhibited low resistance to thiophanate‐methyl, including *C*. *viniferum* (24), *C*. *siamense* (20), *C*. *chrysophilum* (17), *C*. *jiangxiense* (3), *C*. *fructicola* (2), *C*. *theobromicola* (2), *C*. *hebeiense* (1) and *C*. *gloeosporioides* (1). By contrast, 17 isolates were classified as highly resistant, comprising *C*. *siamense* (10), *C*. *viniferum* (4), *C*. *hebeiense* (2) and *C*. *fructicola* (1). Only two isolates were sensitive to the fungicide, one belonging to *C*. *viniferum* and one to *C*. *chrysophilum*. Low‐ and high‐resistance phenotypes were detected across all eight species evaluated (Tables 1 and 2; Supporting Information, Table S1).

**Table 1:** Sensitivity classification of *Colletotrichum gloeosporioides* complex isolates to thiophanate‐methyl

Complex	Species	No. of isolates	Sensitive	Low resistant	Highly resistant
*C*. *gloeosporioides*	*C*. *siamense*	30	0	20	10
	*C*. *viniferum*	29	1	24	4
	*C*. *chrysophilum*	18	1	17	0
	*C*. *fructicola*	3	0	2	1
	*C*. *hebeiense*	3	0	1	2
	*C*. *jiangxiense*	3	0	3	0
	*C*. *theobromicola*	2	0	2	0
	*C*. *gloeosporioides*	1	0	1	0
Total		89	2	70	17

**Table 2:** Amino acid sequence alignment of the partial *β*‐*tubulin* gene for *Colletotrichum siamense*, *C*. *viniferum*, *C*. *hebeiense* and *C*. *chrysophilum* isolates from grape and sensitive (S), low‐resistant (LR) or highly resistant (HR) to thiophanate‐methyl. Amino acids shown in bold represent the nucleotides present at codons 167, 198, and 200

Isolates (MBC phenotype) (GenBank accession number)	Codons																			
				167								198		200						
Sensitive																				
*C*. *siamense* (KM885294)	M	A	T	**F**	…S	Q	L	V	E	N	D	**E**	T	**F**	C	I	D	N	E	A
VlCvPR22_106 (S) (PZ246657)	M	A	T	**F**	…S	Q	L	V	E	N	D	**E**	T	**F**	C	I	D	N	E	A
VlCcBA22_136 (S) (PZ246658)	M	A	T	**F**	…S	Q	L	V	E	N	D	**E**	T	**F**	C	I	D	N	E	A
Low‐resistant																				
VlCvPR22_191 (LR) (PZ246659)	M	A	T	**F**	…S	Q	L	V	E	N	D	**E**	T	**F**	C	I	D	N	E	A
VlCvPR22_187 (LR) (PZ246660)	M	A	T	**F**	…S	Q	L	V	E	N	D	**E**	T	**F**	C	I	D	N	E	A
Highly resistant																				
*C*. *cereale* (FJ476051)	M	A	T	**F**	…S	Q	L	V	E	N	D	**E**	T	**Y**	C	I	D	N	E	A
*C*. *siamense* (KM885297)	M	A	T	**F**	…S	Q	L	V	E	N	D	**A**	T	**F**	C	I	D	N	E	A
VlCsPR22_206 (HR) (PZ246661)	M	A	T	**F**	…S	Q	L	V	E	N	D	**A**	T	**F**	C	I	D	N	E	A
VlCsPR22_198 (HR) (PZ246662)	M	A	T	**F**	…S	Q	L	V	E	N	D	**A**	T	**F**	C	I	D	N	E	A
VlCsPE22_140 (HR) (PZ246663)	M	A	T	**F**	…S	Q	L	V	E	N	D	**A**	T	**F**	C	I	D	N	E	A
VlChRS22_197 (HR) (PZ246664)	M	A	T	**F**	…S	Q	L	V	E	N	D	**E**	T	**Y**	C	I	D	N	E	A

MBC, methyl benzimidazole carbamate; VlCc, isolate of *C*. *chrysophilum* from *Vitis labrusca*; VlCv, isolates of *C*. *viniferum* from *V*. *labrusca*; VlCs, isolate of *C*. *siamense* from *V*. *labrusca*; ClCh, isolate of *C*. *hebeiense* from *V*. *labrusca*.

For azoxystrobin, a total of 143 isolates of *Colletotrichum* spp. were classified as sensitive, and 33 were classified as resistant (Table 1; Supporting Information, Table S1). No isolates were classified as moderately resistant, because all isolates that were inhibited at 3 μg mL^−1^ were also inhibited at 100 μg mL^−1^ of azoxystrobin. However, the classification differed between treatments with and without SHAM at both 3 and 100 μg mL^−1^ of azoxystrobin. A total of 49 isolates were classified as completely resistant in the absence of SHAM, whereas 33 isolates were classified as completely resistant in the presence of SHAM (Table 3). Among the 16 isolates that germinated in the absence of SHAM but were inhibited when SHAM was added, 10 belonged to *C*. *nymphaeae*, 3 to *C*. *siamense*, 1 to *C*. *viniferum* and 1 to *C*. *fructicola*.

**Table 3:** Number of *Colletotrichum* spp. isolates with germination above 95% on medium amended with 3 and 100 μg mL^−1^ azoxystrobin and with or without salicylhydroxamic acid (SHAM)

Complex	Species	Control	Azoxystrobin	Azoxystrobin	Azoxystrobin (100 μg mL^−1^)	Azoxystrobin
			(3 μg mL^−1^)	(3 μg mL^−1^) + SHAM		(100 μg mL^−1^) + SHAM
*C*. *acutatum*	*C*. *nymphaeae*	74	26	16	26	16
*C*. *gloeosporioides*	*C*. *siamense*	30	14	11	14	11
	*C*. *viniferum*	29	2	1	2	1
	*C*. *chrysophilum*	18	3	2	3	2
	*C*. *hebeiense*	3	1	1	1	1
	*C*. *fructicola*	3	2	1	2	1
	*C*. *theobromicola*	2	1	1	1	1
Total		159	49	33	49	33

Considering both fungicides phenotypes, 60 azoxystrobin‐sensitive isolates of the *C*.*g*. complex also showed low resistance to thiophanate‐methyl. Isolates classified as azoxystrobin‐resistant were either considered low‐resistant (ten isolates) or highly resistant (seven isolates) to thiophanate‐methyl. In addition, ten isolates were sensitive to azoxystrobin but highly resistant to thiophanate‐methyl, and only two isolates were sensitive to both fungicides. No isolates were resistant to azoxystrobin while remaining sensitive to thiophanate‐methyl (Fig. 1).

**Figure 1:**
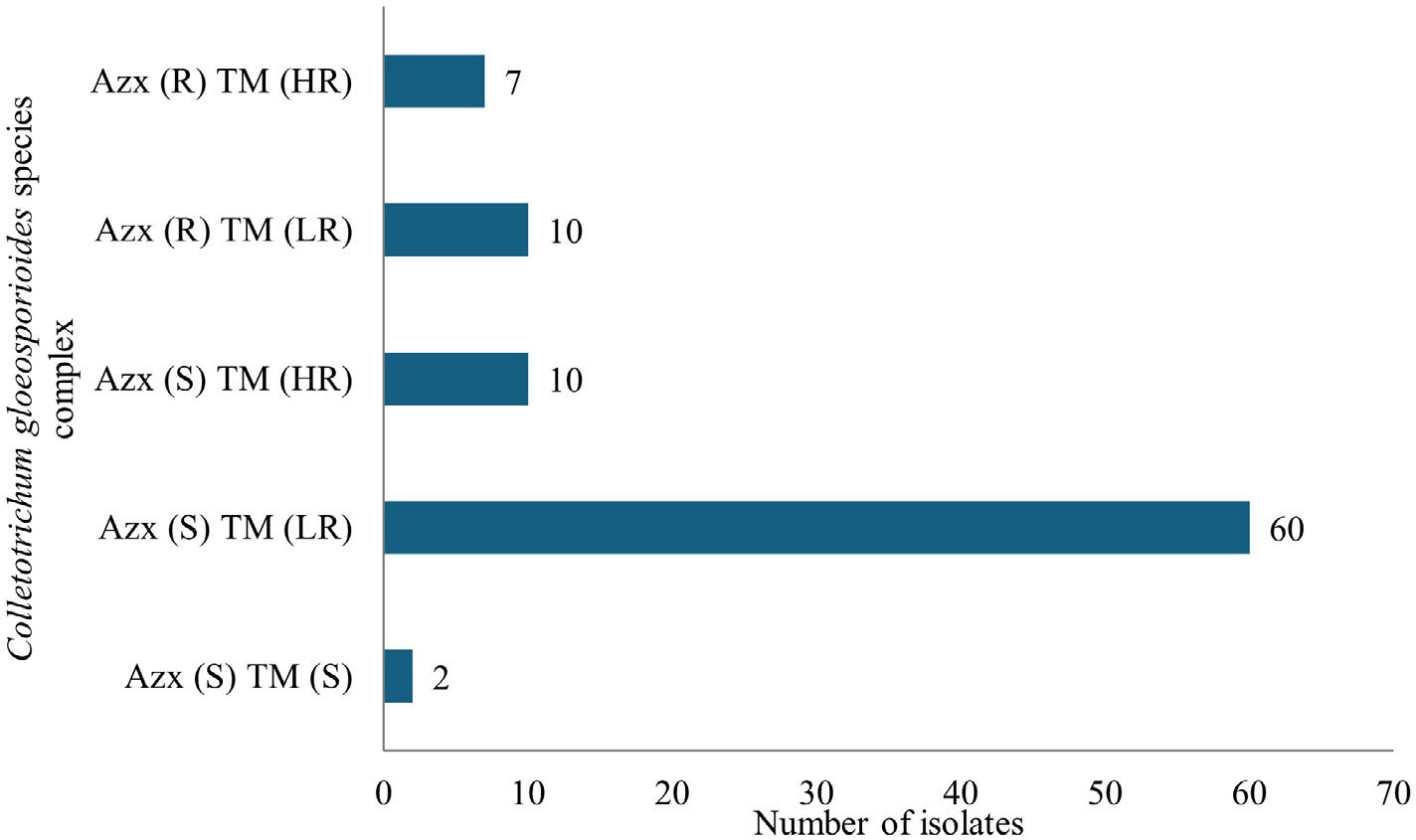
Quinone outside inhibitor (QoI) and methyl benzimidazole carbamate (MBC) fungicide sensitivity phenotypes observed in *Colletotrichum gloeosporioides* complex isolates in Brazil. TM, thiophanate‐methyl; Azx, azoxystrobin. Isolates are labeled as: sensitive (S) low‐resistant (LR), resistant (R) or highly resistant (HR).

### 3.2 Molecular characterization of the *β*‐*tubulin* gene in isolates with different thiophanate‐methyl phenotypes

Three of the four highly resistant isolates exhibited a change from glutamic acid to alanine at codon 198 (E198A); one of the four had a change from phenylalanine to tyrosine at codon 200 (F200Y). Sensitive and low‐resistant isolates did not show mutations at codons 167, 198 and 200, which are often associated with resistant phenotypes (Table 2). The partial *β*‐*tubulin* sequence obtained from isolates VlCvPR22_106 and VlCcBA22_136 (sensitive), VlCvPR22_191 and VlCvPR22_187 (low‐resistant), VlCsPR22_206, VlCsPR22_198, VlCsPE22_140 and VlChRS22_197 (highly resistant) were deposited in GenBank.

### 3.3 Molecular characterization of *cytb* sequences in isolates sensitive and resistant to azoxystrobin fungicides

Amino acid substitutions at codon 143 of the *cytb* gene were detected only in isolates that showed conidial germination at 3 and 100 μg mL^−1^ azoxystrobin in the presence of SHAM (100 μg mL^−1^). Of the 49 isolates that germinated at the same azoxystrobin concentrations in the absence of SHAM, the 16 isolates whose germination was inhibited by the addition of SHAM did not carry mutations at codons 129, 139, or 143. These results indicate that resistance identified in treatments without SHAM included false positives. No amino acid variation was observed among QoI‐sensitive isolates.

The *cytb* gene from six *C*. *nymphaeae* isolates with varying response to azoxystrobin was sequenced. Isolate VvCnPR22_118 germinated only in the non‐fungicide‐treated controls. Isolates VlCnPR22_180 and VlCnPR22_230 showed 38% and 40% germination at 3 μg mL^−1^ azoxystrobin without SHAM, and 23% and 44% at 100 μg mL^−1^ without SHAM. No germination occurred for these isolates in treatments with azoxystrobin and SHAM. These isolates had no mutations and were classified as sensitive. Isolates VvCnPR22_217, VvCnRS22_110 and VlCnPR22_86 showed more than 95% germination at 3 and 100 μg mL^−1^ azoxystrobin with SHAM, and mutations at codon 143 were detected, classifying them as resistant (Table 4).

**Table 4:** Partial amino acid sequences of the cytochrome *b* protein from *Colletotrichum nymphaeae*, *C*. *siamense*, *C*. *fructicola*, *C*. *viniferum* and *C*. *chrysophilum* isolates sensitive (S) or resistant (R) to azoxystrobin. Amino acids shown in bold represent the nucleotides present at codons 129, 139, and 143

	Codons														
Isolates (QoI phenotype) (GenBank accession number)	129									139				143	
Reference isolates															
*C*. *siamense* (KX885103) (S)	**F**	L	G	.	.	P	Y	G	Q	**M**	S	L	.	**G**	**T**
*C*. *siamense* (KM885303) (R)	**F**	L	G	.	.	P	Y	G	Q	**M**	S	L	.	**A**	**T**
*C*. *acutatum* (MF784586.1) (MR^†^)	**L**	L	G	.	.	P	Y	G	Q	**M**	S	L	.	**G**	**T**
*C*. *acutatum* (MF784587.1) (R)	**F**	L	G	.	.	P	Y	G	Q	**M**	S	L	.	**A**	**T**
*C*. *nymphaeae*															
VvCnPR22_118 (S) (PZ255810)	**F**	L	G	.	.	P	Y	G	Q	**M**	S	L	.	**G**	T
VlCnPR22_180 (S) (PZ255809)	**F**	L	G	.	.	P	Y	G	Q	**M**	S	L	.	**G**	T
VlCnPR22_230 (S) (PZ255814)	**F**	L	G	.	.	P	Y	G	Q	**M**	S	L	.	**G**	T
VvCnPR22_217 (R) (PZ255811)	**F**	L	G	.	.	P	Y	G	Q	**M**	S	L	.	**A**	T
VvCnRS22_110 (R) (PZ255812)	**F**	L	G	.	.	P	Y	G	Q	**M**	S	L	.	**A**	T
VlCnPR22_86 (R) (PZ255813)	**F**	L	G	.	.	P	Y	G	Q	**M**	S	L	.	**A**	T
*C*. *siamense*															
VlCsPR22_232 (S) (PZ255800)	**F**	L	G	.	.	P	Y	G	Q	**M**	S	L	.	**G**	T
VvCsBR22_57 (S) (PZ255801)	**F**	L	G	.	.	P	Y	G	Q	**M**	S	L	.	**G**	T
VvCsBR22_60 (S) (PZ255797)	**F**	L	G	.	.	P	Y	G	Q	**M**	S	L	.	**G**	T
VvCsBR22_59 (S) (PZ255798)	**F**	L	G	.	.	P	Y	G	Q	**M**	S	L	.	**G**	T
VvCsBR22_64 (S) (PZ255799)	**F**	L	G	.	.	P	Y	G	Q	**M**	S	L	.	**G**	T
VlCsPR22_207 (R) (PZ255802)	**F**	L	G	.	.	P	Y	G	Q	**M**	S	L	.	**A**	T
VlCsPR22_206 (R) (PZ255803)	**F**	L	G	.	.	P	Y	G	Q	**M**	S	L	.	**A**	T
VlCsPR22_194 (R) (PZ255804)	**F**	L	G	.	.	P	Y	G	Q	**M**	S	L	.	**A**	T
*C*. *chrysophilum*															
VlCcPR22_49 (S) (PZ261635)	**F**	L	G	.	.	P	Y	G	Q	**M**	S	L	.	**G**	T
VlCcBA22_136 (S) (PZ261636)	**F**	L	G	.	.	P	Y	G	Q	**M**	S	L	.	**G**	T
VlCcPR22_44 (S) (PZ261637)	**F**	L	G	.	.	P	Y	G	Q	**M**	S	L	.	**G**	T
VlCcRS22_54 (R) (PZ255805)	**F**	L	G	.	.	P	Y	G	Q	**M**	S	L	.	**A**	T
VlCcPR22_153 (R) (PZ255806)	**F**	L	G	.	.	P	Y	G	Q	**M**	S	L	.	**A**	T
*C*. *viniferum*															
VlCvPR22_186 (S) (PZ261638)	**F**	L	G	.	.	P	Y	G	Q	**M**	S	L	.	**G**	T
VlCvPR22_107 (S) (PZ261639)	**F**	L	G	.	.	P	Y	G	Q	**M**	S	L	.	**G**	T
VlCvPR22_99 (S) (PZ261640)	**F**	L	G	.	.	P	Y	G	Q	**M**	S	L	.	**G**	T
VlCvPR22_191 (S) (PZ261641)	**F**	L	G	.	.	P	Y	G	Q	**M**	S	L	.	**G**	T
VlCvPE22_171 (R) (PZ255808)	**F**	L	G	.	.	P	Y	G	Q	**M**	S	L	.	**A**	T
Isolates from *C*. *fructicola*															
VvCfBR22_61 (S) (PZ261642)	**F**	L	G	.	.	P	Y	G	Q	**M**	S	L	.	**G**	T
VlCfPR22_163 (R) (PZ255807)	**F**	L	G	.	.	P	Y	G	Q	**M**	S	L	.	**A**	T

MR, moderately resistant; QoI, quinone outside inhibitor; VlCc, isolate of *C*. *chrysophilum* from *Vitis labrusca*; VlCv, isolates of *C*. *viniferum* from *V*. *labrusca*; VlCs, isolate of *C*. *siamense* from *V*. *labrusca*; VvCs, isolate of *C*. *siamense* from *V*. *vinifera*; VvCn, isolate of *C*. *nymphaeae* from *V*. *vinifera*; VlCn, isolate of *C*. *nymphaeae* from *V labrusca*; VlCf, isolate of *C*. *fructicola* from *V*. *labrusca*.

^†^ MR: moderately resistant.

For isolates belonging to the *C*.*g*. complex, the *cytb* gene was also sequenced. *C*. *siamense* isolate VvCsBR22_60 germinated only in the controls. Isolates VlCsPR22_232, VvCsBR22_57, VvCsBR22_59 and VvCsBR22_64 showed 63%, 55%, 59% and 45% germination at 3 μg mL^−1^ azoxystrobin without SHAM, and 30%, 47%, 64% and 38% at 100 μg mL^−1^ azoxystrobin without SHAM, respectively. No germination was observed in treatments of azoxystrobin with SHAM. These isolates showed no mutations and were classified as sensitive. Isolates VlCsPR22_207, VlCsPR22_206 and VlCsPR22_194 exhibited more than 95% germination in treatments of azoxystrobin with SHAM at both doses, with mutations detected at codon 143, classifying them as resistant. (Table 4).

*Colletotrichum chrysophilum* isolate VlCcPR22_44 germinated only in unamended medium. Isolates VlCcPR22_49 and VlCcBA22_136 showed 39% and 83% germination at 3 μg mL^−1^ azoxystrobin without SHAM, and 22% and 52% at 100 μg mL^−1^ without SHAM, respectively. No germination was observed in azoxystrobin with SHAM. These isolates had no mutations in *cytb* and were classified as sensitive. Isolates VlCcRS22_54 and VlCcPR22_153 exhibited more than 95% germination in the treatments azoxystrobin with SHAM at both doses, with mutations at codon 143, classifying them as resistant (Table 4).

*Colletotrichum viniferum* isolate VlCvPR22_99 germinated only in unamended medium. Isolates VlCvPR22_186, VlCvPR22_107 and VlCvPR22_191 showed 25%, 5% and 36% germination at 3 μg/mL azoxystrobin without SHAM, and 19%, 0% and 28% at 100 μg/mL without SHAM, respectively. No germination was observed in azoxystrobin with SHAM treatments. These isolates had no mutations in the *cytb* gene and were classified as sensitive. Isolate VlCvPE22_171 exhibited more than 95% germination in treatments with azoxystrobin and SHAM at both doses, with mutations at codon 143, classifying them as resistant (Table 4).

*Colletotrichum fructicola* isolate VvCfBR22_61 showed 80% and 76% germination at 3 and 100 μg mL^−1^ azoxystrobin without SHAM, respectively. No germination was observed in the azoxystrobin with SHAM treatments. This isolate had no mutations and was classified as sensitive. Isolate VlCfPR22_163 showed more than 95% germination in the azoxystrobin with SHAM treatments, with mutations at codon 143, classifying it as resistant (Table 4).

### 3.4 Detached fruit studies

Label rates of commercial formulations of azoxystrobin and thiophanate‐methyl failed to control GRR on berries inoculated with QoI‐resistant isolates and with MBC low‐ and high‐resistant isolates of *Colletotrichum* spp. In the first trial, GRR incidence on treated berries ranged from 33% to 100%, whereas in the second trial incidence ranged from 56% to 100% for both fungicides (Tables 5 and 6). By contrast, label rates effectively controlled the disease on berries inoculated with sensitive isolates.

**Table 5:** Grape ripe rot incidence caused by *Colletotrichum* spp. isolates sensitive (S) or resistant (R) to azoxystrobin on injured or non‐injured fruit treated with Amistar WG®

Complex	Grape ripe rot incidence (%)							
				Experiment 1				Experiment 2			
	Specie	Isolates	Phenotype	With wound		Without wound		With wound		Without wound	
				Untreated	Treated	Untreated	Treated	Untreated	Treated	Untreated	Treated
*C*. *gloeosporioides*	*C*. *fructicola*	VvCfBR22‐61	S	67	0	0	0	100	0	0	0
		**VlCfpr22‐163** ^**†**^	R	100	100	0	0	100	100	0	0
*C*. *gloeosporioides*	*C*. *hebeiense*	VlChRS22‐55	S	67	0	0	0	78	0	0	0
		**VlChRS22‐75** ^**†**^	R	100	78	0	0	56	100	0	0
*C*. *gloeosporioides*	*C*. *viniferum*	VlCvPR22‐186	S	78	0	0	0	78	0	11	0
		VlCvPR22‐99	S	100	0	0	0	100	0	0	0
		**VlCvPE22‐171**	R	67	67	0	0	100	100	22	22
*C*. *acutatum*	*C*. *nymphaeae*	VlCnPR22‐180	S	100	0	0	0	67	0	0	0
		VvCnPR22‐119	S	100	0	0	0	67	0	0	0
		**VvCnRS22‐110**	R	100	100	0	0	100	100	0	0
		**VvCnPR22‐210**	R	100	100	0	0	67	56	22	11
*C*. *gloeosporioides*	*C*. *chrysophilum*	VlCcPR22‐44	S	100	0	0	0	89	0	0	0
		VlCcPR22‐49	S	56	0	0	0	100	0	0	0
		**VlCcRS22‐54**	R	78	67	0	0	56	67	11	0
		**VlCcPR22‐153**	R	56	78	0	0	56	78	11	22
*C*. *gloeosporioides*	*C*. *siamense*	VvCsBR22‐59	S	78	0	0	0	89	0	22	0
		VvCsBR22‐57	S	67	0	0	0	100	0	0	0
		**VlCsPR22‐194** ^**†**^	R	100	78	0	0	100	67	0	0
		**VlCsPR22‐202** ^**†**^	R	100	67	0	0	67	78	11	11
		**VlCsPR22‐204** ^**†**^	R	56	78	0	0	56	78	0	0
		**VlCsPR22‐205** ^**†**^	R	67	67	0	0	78	100	11	0
		**VlCsPR22‐206** ^**†**^	R	78	67	0	0	100	100	0	0

^†^ Isolate were resistant to azoxystrobin and highly resistant to thiophanate‐methyl. Resistant isolates are shown in bold.

**Table 6:** Grape ripe rot incidence caused by *Colletotrichum* spp. isolates classified as sensitive (S), low‐resistant (LR), and highly resistant (HR) to thiophanate‐methyl on injured and non‐injured fruit treated with Cercobin 700 WG

				Grape ripe rot incidence (%)							
				Experiment 1				Experiment 2			
				With wound		Without wound		With wound		Without wound	
Complex	Specie	Isolates	Phenotype	Untreated	Treated	Untreated	Treated	Untreated	Treated	Untreated	Treated
*C*. *gloeosporioides*	*C*. *theobromicola*	VlCtPR22_195	LR	100	67	0	0	100	100	100	100
	*C*. *fructicola*	**VlCfpr22_163** ^**†**^	HR	33	33	0	0	56	67	0	0
	*C*. *chrysophilum*	VlCcBA22_136	S	56	0	0	0	100	0	0	0
		VlCcPR22_41	LR	100	56	0	0	100	44	0	0
	*C*. *hebeiense*	VlChRS22_55	LR	33	33	0	0	100	56	0	0
		**VlChRS22_75** ^**†**^	HR	67	56	0	0	67	67	0	0
		**VlChRS22_197**	HR	44	33	0	0	56	67	0	0
	*C*. *viniferum*	VlCvPR22_106	S	89	0	0	0	100	0	0	0
		VlCvPR22_187	LR	100	100	0	0	78	100	0	0
		VlCvPE22_167	LR	100	100	0	0	100	56	0	0
		**VlCvPR22_96**	HR	78	67	0	0	100	100	0	0
		**VlCvPR22_97**	HR	67	33	0	0	100	100	0	0
	*C*. *siamense*	VvCsBR22_57	LR	100	44	0	0	100	22	0	0
		**VlCsPR22_194** ^**†**^	HR	67	56	0	0	78	89	0	0
		**VlCsPR22_202** ^**†**^	HR	67	67	0	0	78	67	0	0
		**VlCsPR22_204** ^**†**^	HR	89	78	0	0	67	78	0	0
		**VlCsPR22_205** ^**†**^	HR	100	56	0	0	78	67	0	0
		**VlCsPR22_206** ^**†**^	HR	100	100	0	0	100	100	0	0
		**VlCsPE22_165**	HR	100	89	0	0	100	89	89	11

^†^ Isolate were resistant to azoxystrobin and highly resistant to thiophanate‐methyl. Highly resistant isolates are shown in bold.

Five *C*. *siamense* isolates, one *C*. *fructicola* isolate and one *C*. *hebeiense* isolate were resistant to azoxystrobin and highly resistant to thiophanate‐methyl (Tables 5 and 6).

## 4 DISCUSSION

In this study, *C*.*g*. complex isolates exhibited varying levels of sensitivity to thiophanate‐methyl. Most isolates were classified as low‐resistant (78.6%), whereas 19.1% were highly resistant. High resistance in our study was associated with amino acid changes at codons 198 and 200 in *β*‐*tubulin*, consistent with prior reports.^18^, ^32^ Genotypes such as E198A and E198K have been documented in multiple fungi, including the *C*.*g*. complex, *Venturia inaequalis*, *Botrytis cinerea* and others.^33^, ^34^, ^35^, ^36^, ^37^ Low‐resistant isolates are often associated with amino acid changes at codon 167 (F167Y).^16^ However, in this study, low‐resistant isolates lacked the F167Y mutation, contrasting with previous reports in which low‐resistant *Colletotrichum* isolates carried this mutation. In another study, low‐resistant isolates of *Monilinia fructicola* were also able to cause disease on thiophanate‐methyl treated peaches, but no mutations in the *β‐tubulin* gene were identified to explain this outcome.^38^ The mechanism of resistance in our study or the aforementioned study in low‐resistant isolates is unknown but may be linked to target gene overexpression or the activity of ATP‐binding cassette (ABC) transporters, which can reduce fungicide accumulation in the pathogen mycelium and confer partial insensitivity.^39^

The ability of low‐resistant isolates to generate disease on fruit treated with the label rate of formulated thiophanate‐methyl was unexpected. With few exceptions, low‐resistant isolates caused GRR on only injured and not uninjured fruit under our experimental conditions. Therefore, thiophanate‐methyl may still be effective in spray programs to manage sensitive and low‐resistant isolates of *Colletotrichum* species that are intrinsically sensitive to this fungicide if wounding is minimized, through insect pest management for example. Based on previous phylogenetic analyses,^1^ it is possible to conclude that the Southern region of Brazil shows a high frequency of species belonging to the *C*.*a*. complex associated with grapevine, which may compromise the efficacy of thiophanate‐methyl in this region, although the most isolates were isolated from endophytic tissue.^1^ By contrast, in the Northeastern region of Brazil, only species in the *C*.*g*. complex were identified.^1^ Under these conditions, use of thiophanate‐methyl may be recommended in association or alternation with different fungicides. However, sensitivity monitoring studies should be continuously conducted to track the potential selection of resistant isolates in the region and also to understand the selection pressure under endophytic population.

Isolates of the *C*.*a*. complex were not included in the thiophanate‐methyl sensitivity assays, because this species is known to be inherently insensitive to MBC fungicides.^19^ For example, another study observed no point mutations in the *β‐tubulin* gene of resistant *C*.*a*. complex isolates, concluding that insensitivity to MBC fungicides is intrinsic to this species.^40^ Similar findings have been reported for *C*.*a*. complex isolates from citrus and strawberry in Brazil.^41^, ^42^ One possible explanation for this intrinsic tolerance is the *CaABC1* gene, which encodes an ABC transporter potentially involved in tolerance to abiotic stress and fungicide resistance in *C*.*a*. complex.^43^

The intrinsic risk of resistance development to QoI fungicides is high,^44^ and our study identified the presence of resistance to azoxystrobin in 33 isolates from *C*.*a*. and *C*.*g*. complexes. Similar results were observed in other studies reporting that isolates of the *C*.*a*. and *C*.*g*. complexes were resistant to QoI fungicides.^22^, ^23^, ^45^, ^46^ Resistant isolates germinated at 3 and 100 μg mL^−1^ azoxystrobin.^22^ Thus, a discriminatory dose of 3 μg mL^−1^ with SHAM effectively distinguished between sensitive and highly resistant *Colletotrichum* isolates. QoI fungicides target the cytochrome *bc*_1_ complex, and changes at codon 143 in *cytb* gene (G143A) confer high resistance to azoxystrobin,^47^ whereas changes at codons 129 and 137 (F129L and G137R) are linked to moderate resistance.^22^, ^28^ In our study, isolates that germinated at 3 and 100 μg mL^−1^ with SHAM (100 μg mL^−1^) were classified as highly resistant and carried the G143A amino acid change. These isolates also caused GRR symptoms on grapes treated with azoxystrobin at the label rate.

The results of our study contrast sharply with earlier findings suggesting that the use of SHAM was unnecessary to characterize azoxystrobin sensitivity in *Colletotrichum* isolates from the *C*.*a*. and *C*.*g*. complexes.^22^, ^28^ In our study, treatment with azoxystrobin in the presence of SHAM prevented false positives, allowing the identification of resistant isolates with the mutation in the *cytb* gene, even though the same methodology as in the cited studies was used. The need for SHAM to distinguish QoI‐resistant from QoI‐sensitive isolates is consistent with previous literature highlighting the key role of SHAM in obtaining more accurate effective concentration values resulting in 50% inhibition (EC_50_) for QoI fungicides in the *C*.*g*. complex from *Citrus sinensis*.^48^ The authors reported that the addition of SHAM reduced the variability of EC_50_ data, leading to a more precise assessment of isolate sensitivity to azoxystrobin. The method used in their study, inhibition of spore germination, was the same as that used in our research. Furthermore, the importance of SHAM for accurate sensitivity assessment has also been demonstrated in other fungal pathogens, such as *M*. *fructicola*.^49^

Whether recent GRR outbreaks in Northeastern Brazil is linked to emerging fungicide resistance is still unclear but this study does provide preliminary support for this hypothesis. Specifically, 2 of 14 isolates were resistant to azoxystrobin, whereas 5 of 14 exhibited resistances to thiophanate‐methyl. Moreover, seven isolates showed dual resistance to both fungicides, including five *C*. *siamense*, one *C*. *fructicola* and one C. *hebeiense*. Dual resistance has been reported in other crops,^20^ ^,^ ^50^ but its occurrence in grapevine pathogens underscores the urgency of resistance monitoring and implementation of resistance management strategies.

## 5 CONCLUSION

To our knowledge, this is the first report demonstrating resistance to both QoI and MBC fungicides in *Colletotrichum* isolates associated with grapevine in Brazil. Further research is needed to investigate the sensitivity of *Colletotrichum* isolates to other active ingredients or chemical groups, as well as to explore alternative strategies for the sustainable management of the disease in the field.

## CONFLICT OF INTEREST

The authors declare that they do not have any competing interests.

## AUTHOR CONTRIBUTIONS

DRMS, CIZ, JG, GS and LLMDM conceived and designed research. DRMS, CZ, DF and GJ conducted experiments. DRMS and JG analyzed the data. DRMS, JG, GS and LLMDM wrote the manuscript, the other authors provided feedback, and all authors approved the manuscript.

## Supporting information


**Table S1.** QoI and MBC fungicide sensitivity phenotypes observed in *Colletotrichum* species complex by Brazilian States.

## Data Availability

The data that support the findings of this study are available from the corresponding author upon reasonable request.
