# Supplementary material for: Resistance to thiophanate‐methyl and azoxystrobin in Colletotrichum species from Brazilian grapes
Source: Pest Manag Sci. 2026 Aug 17;82(12):12121–30. doi: 10.1002/ps.71203 (PMC13756573; doi:10.1002/ps.71203)
Supplement: Supplementary file 1 [file PS-82-12121-s001.docx]

Table S1: QoI and MBC fungicide sensitivity phenotypes observed in *Colletotrichum* species complex by Brazilian States.

| State | Species | Azoxystrobin | | Thiophanate methyl | | |
| --- | --- | --- | --- | --- | --- | --- |
|  |  | Sensitive | Resistant | Sensitive | Low-resistant | High resistant |
| Rio Grande do Sul | *C. nymphaeae* | 2 | 1 | N/A | N/A | N/A |
|  | *C. chrysophilum* | 1 | 1 | N/A | 2 | N/A |
|  | *C. hebeiense* | N/A* | 1 | N/A | 1 | N/A |
| Paraná | *C. nymphaeae* | 57 | 14 | N/A | N/A | N/A |
|  | *C. viniferum* | 22 | N/A | 1 | 18 | 3 |
|  | *C. siamense* | 8 | 10 | N/A | 12 | 6 |
|  | *C. chrysophilum* | 13 | 1 | N/A | 14 | N/A |
|  | *C. melonis* | 8 | N/A | N/A | N/A | N/A |
|  | *C. cf. melonis* | 5 | N/A | N/A | N/A | N/A |
|  | *C. fructicola* | N/A | 1 | N/A | N/A | 1 |
|  | *C. hebeiense* | 1 | 1 | N/A | N/A | 2 |
|  | *C. jiangxiense* | 3 | N/A | N/A | 3 | N/A |
|  | *C. gloeosporioides* | 1 | N/A | N/A | 1 | N/A |
|  | *C. theobromicola* | 1 | 1 | N/A | 2 | N/A |
| São Paulo | *C. chrysophilum* | 1 | N/A | N/A | 1 | N/A |
| Distrito Federal | *C. siamense* | 6 | N/A | N/A | 6 | N/A |
|  | *C. fructicola* | 2 | N/A | N/A | 2 | N/A |
| Pernanbuco | *C. viniferum* | 6 | 1 | N/A | 6 | 1 |
|  | *C. siamense* | 4 | 1 | N/A | 2 | 3 |
| Bahia | *C. chrysophilum* | 1 | N/A | 1 | N/A | N/A |
|  | *C. siamense* | 1 | N/A | N/A | N/A | 1 |
| Total |  | 143 | 33 | 2 | 70 | 17 |

* (N/A): not available
